# Spectroscopic Ellipsometry Characterization of As-Deposited and Annealed Non-Stoichiometric Indium Zinc Tin Oxide Thin Film

**DOI:** 10.3390/ma14030578

**Published:** 2021-01-26

**Authors:** Petr Janicek, Maryane Putri, Ki Hwan Kim, Hye Ji Lee, Marek Bouska, Stanislav Šlang, Hee Young Lee

**Affiliations:** 1 Institute of Applied Physics and Mathematics, Faculty of Chemical Technology, University of Pardubice, Studentska 95, 53210 Pardubice, Czech Republic; petr.janicek@upce.cz; 2 Center of Materials and Nanotechnologies, Faculty of Chemical Technology, University of Pardubice, Studentska 95, 53210 Pardubice, Czech Republic; marek.bouska@upce.cz (M.B.); stanislav.slang@upce.cz (S.Š.); 3 School of Materials Science and Engineering, Yeungnam University, 280 Daehak-Ro, Gyeongsan, Gyeongbuk 38541, Korea; maryane.putri@gmail.com (M.P.); skyever88@gmail.com (K.H.K.); lhj7446@gmail.com (H.J.L.); 4 Department of Graphic Arts and Photophysics, Faculty of Chemical Technology, University of Pardubice, Studentska 95, 53210 Pardubice, Czech Republic

**Keywords:** optical properties, spectroscopic ellipsometry, RF magnetron sputtering, indium zinc tin oxide, non-stoichiometric

## Abstract

A spectroscopic ellipsometry study on as-deposited and annealed non-stoichiometric indium zinc tin oxide thin films of four different compositions prepared by RF magnetron sputtering was conducted. Multi-sample analysis with two sets of samples sputtered onto glass slides and silicon wafers, together with the analysis of the samples onto each substrate separately, was utilized for as-deposited samples. Annealed samples onto the glass slides were also analyzed. Spectroscopic ellipsometry in a wide spectral range (0.2–6 eV) was used to determine optical constants (refractive index *n* and extinction coefficient *k*) of these films. Parameterized semiconductor oscillator function, together with Drude oscillator, was used as a model dielectric function. Geometrical parameters (layer thickness and surface roughness) and physical parameters (direct optical bandgap, free carrier concentration, mobility, and specific electrical resistivity) were determined from spectroscopic ellipsometry data modeling. Specific electrical resistivity determined from the Drude oscillator corresponds well with the results from electrical measurements. Change in the optical bandgap, visible especially for annealed samples, corresponds with the change of free carrier concentration (Moss–Burstein effect). Scanning electron microscope did not reveal any noticeable annealing-induced change in surface morphology.

## 1. Introduction

For utilization in flat panel displays, transparent conductive oxides have been used for many years due to their low resistivity and high transparency in the visible spectrum (e.g., References [1,2,3]). Indium tin oxide (ITO) is used most widely for transparent conducting films because of its low resistivity (~1 × 10^−4^ Ω⋅cm), high optical transmittance ~85%, and wide optical bandgap of approximately 3 eV (e.g., References [4,5,6]).

Refractive index and extinction coefficient of ITO were intensively studied in the past, including spectroscopic ellipsometry as a tool for the determination of these two physical quantities (e.g., References [7,8,9,10]). Different model dielectric functions were used for evaluation of data from spectroscopic ellipsometry, including Drude oscillator [7,8,10], single oscillator [7], a combination of Lorentz-type oscillators [8], and a new amorphous model [9].

Indium-saving oxide, i.e., indium zinc tin oxide (IZTO), thin films have been investigated as possible electrodes for optoelectronic devices, owing to their high optical transparency, low electrical resistivity, and high work function value (e.g., References [11,12,13,14,15]). IZTO can also lower the price of these devices, since indium is a very expensive raw material due to the limited amount of worldwide deposits.

Properties of non-stoichiometric IZTO films deposited at room temperature, as well as annealed films, including the study of their electrical properties and measurement of optical transmittance and example of utilization of these films in organic photovoltaic, have been reported earlier [16,17,18].

The wide spectral dependence of optical constants (refractive index, *n*, and extinction coefficient, *k*) together with layer thicknesses and surface roughness for as-deposited and annealed non-stoichiometric IZTO thin films with 5% cation excess of either Zn or Sn from nominal stoichiometry (In_2−2x_Zn_x_Sn_x_O_3_), prepared by RF magnetron sputtering obtained by using spectroscopic ellipsometry in the wide spectral range (0.2–6 eV), is presented for the first time. Our manuscript shows procedure how specific electrical conductivity can be determined without the necessity of contacting the samples with quite good comparison with the results from electrical measurement. Utilization of infrared ellipsometry for these samples (neither stoichiometric nor non-stoichiometric IZTO) has not been published previously. Measurement in a wide spectral range also allows for the determination of the direct energy bandgap, which is usually measured by transmission only. The effect of composition and annealing on the properties of the films is also discussed. To our knowledge, the spectroscopic ellipsometry study of non-stoichiometric IZTO layers has not been reported to date.

## 2. Materials and Methods

Sintered ceramic IZTO targets of 3.8 cm in diameter and about 5 mm thickness, with nominal compositions corresponding to In_0.5_Zn_0.20_Sn_0.30_O_1.5_ (Sn-rich IZTO25), In_0.5_Zn_0.30_Sn_0.20_O_1.5_ (Zn-rich IZTO25), In_0.4_Zn_0.25_Sn_0.35_O_1.5_ (Sn-rich IZTO30), and In_0.4_Zn_0.35_Sn_0.25_O_1.5_ (Zn-rich IZTO30), were prepared by using the conventional mixed-oxide method process prior to thin film deposition. The powder mixture of In_2_O_3_, ZnO, and SnO_2_ powders with purities of 99.9% or higher were weighed and mixed according to the chemical compositions of interest. The mixture was then granulated by sieving, and then pressed into the discs by uniaxial pressing. Sintering was usually performed at 1550~1600 °C, in flowing oxygen (for more details, see, e.g., References [1,16]). Non-stoichiometric IZTO thin films were then sputter-deposited onto 15 mm × 15 mm square commercial glass substrate and also onto single crystal Si substrate with 100 nm–thick thermally grown SiO_2_ layer. Deposition of non-stoichiometric IZTO films (labeled as as-deposited) was conducted, using RF magnetron sputtering, at room temperature, for 25 min, using Ar plasma at a working pressure of 5 mTorr. The diameter of the cathode was 3.8 cm, and it was located downward 41 degrees off the vertical axis of the rotating substrate holder with 5.5 cm in diameter. The cathode–substrate distance was maintained at 10 cm, and RF power of 125 W at 13.56 MHz was supplied to the sputtering cathode during sputtering. Prior to deposition, the vacuum chamber was evacuated to a base pressure of 2 × 10^−5^ Torr or below. During the deposition, argon acted as ambient gas, while the gas flow rate was fixed at 20 sccm (standard cubic centimeter per minute). In order to study the influence of annealing treatment, the films of different compositions were annealed at a temperature of 400 °C, in an argon atmosphere, for 30 min, using rapid thermal annealing (labeled as a = annealed).

Two variable angle spectroscopic ellipsometers (VASE and IR-VASE, J. A. Woollam Co., Lincoln, NE, USA) were used for the optical characterization of the prepared samples. The first ellipsometer was equipped with an automatic rotating analyzer over the spectral range 210 nm–1700 nm (UV–VIS–NIR), measuring 30 revolutions with photon energy steps of 0.05 eV at three selected angles of incidence (AOI) (50°, 60°, and 70°). The second ellipsometer was equipped with a rotating compensator for 1.7–6.2 µm (NIR–MIR), using the same AOI, measuring 25 scans and 15 spectra per revolution with wavenumber steps 8 cm^−1^. Near normal incidence optical reflectance and optical transmittance were measured by the same instruments. WVASE32 software was used for the evaluation of the measured data.

The electrical properties of the as-deposited and annealed non-stoichiometric IZTO films were characterized by using Hall effect measurement (Ecopia HMS-5000, Anyang, Korea) and I–V measurement (Keithley 4200-SCS, Beaverton, OR, USA).

The surface topography was characterized by atomic force microscopy (AFM, Solver NEXT, NT-MDT) in semicontact mode. Two measurements (spot size 5 × 5 μm) were performed on each sample, and the average root mean square (RMS) of the surface roughness was determined according to the ISO 4287/1. The scanning electron microscope (SEM) scans and elemental concentration of studied samples were obtained by using a scanning electron microscope LYRA 3 (Tescan, Brno, Czech Republic) equipped with an energy-dispersive X-ray spectroscopy (EDS) analyzer AZtec X-Max 20 (Oxford Instruments, Abingdon, UK), using acceleration voltage 5 kV.

## 3. Results and Discussion

### 3.1. Structure Model and Model Dielectric Function

In this study, two sets of samples of non-stoichiometric indium zinc tin oxide (IZTO) thin films were prepared by RF magnetron sputtering, on a glass substrate and on a silicon substrate with a thermally grown SiO_2_ layer. These two sets of samples were modeled with (i) a semi-infinite glass substrate (optical constants measured separately), (ii) a homogeneous, isotropic layer representing the non-stoichiometric IZTO film, (iii) surface roughness, and (iv) air as the ambient medium and analogously for silicon substrate with SiO_2_ layer (optical constants adopted from Reference [19]).

As the IZTO films exhibit direct optical transition [20], the parameterized semiconductor oscillator function (PSEMI) model, which analytically describes the dielectric functions as the summation of several energy-bounded Gaussian-broadened polynomials and poles, accounting for index effects due to absorption occurring outside the region being modeled, was used (*ε*_(PSEMI)_) [21,22,23].

In the MIR region (0.05 < *E* < 0.5 eV), a simple Drude oscillator was used to model the free carrier absorption (*ε*_(DRUDE)_) [24]. The total model dielectric function, *ε*, used for the non-stoichiometric IZTO films was as follows:

*ε* = ε_(PSEMI)_ + *ε*_(DRUDE)_. (1)

Bruggeman-type effective medium approximation [25] with 50% of voids and 50% of IZTO was used to model surface roughness. To test the influence of the substrate on the optical constants, different approaches were used. Firstly, data from the measurement of the sample deposited onto Si substrate and sample deposited onto glass substrate were analyzed separately. Secondly, multi-sample analysis was employed with simultaneous use of samples sputtered onto the glass substrate and the silicon substrate in the fitting procedure.

Spectroscopic ellipsometry is an indirect optical characterization method, where the measured values *Ψ*_exp_ and Δ_exp_ are compared to the values calculated from the model. Two parameters, amplitude ratio, *Ψ*, and phase shift, Δ, are defined by using the Fresnel reflection coefficients for *p*- (*r*_p_) and *s*- (*r*_s_) polarized light and express the change of the polarization state:

*(r*_p_/*r*_s_*)* = tan(*ψ*)·exp(iΔ). (2)

Ellipsometry data measured in the spectral range from 0.2 to 6 eV for all three angles of incidence, namely 50°, 60°, and 70°, were used in the fit together, simultaneously, with data from optical reflectivity and optical transmission. Quality of the fit is expressed by mean square error (MSE)—see, e.g., Equation (2) in Reference [26]. A more detailed description of the used methodology can be found in our previous works (e.g., References [26,27]).

It is worth mentioning that fit in a wide spectral range has, overall, 14 free parameters (including thicknesses), and therefore correlations among these parameters are very likely. When conducting fit, it is important to control this (e.g., compare the resulting value of each parameter among all samples). Moreover, the validity of obtained results should be compared with direct methods if possible.

**Figure 1:**
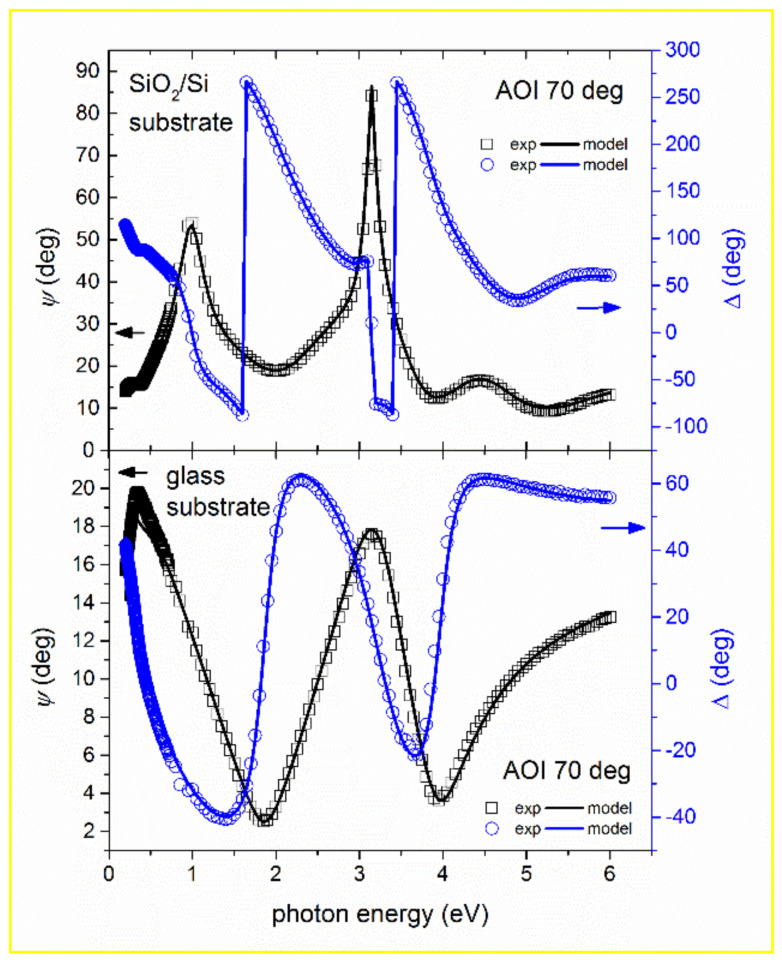
Measured values of *ψ* (squares) and Δ (circles) as a function of photon energy, *E*, for In_0.4_Zn_0.25_Sn_0.35_O_1.5_ (Sn-rich indium zinc tin oxide (IZTO)30) sample in a wide spectrum for samples as-deposited onto SiO_2_/Si substrate (upper part) and onto glass substrate (lower part). The best fit for the angle of incidence 70° is shown by solid lines.

Overall sufficient fit quality of the ellipsometry (*Ψ* and Δ) and transmission and reflectivity data were obtained (see Figure 1 as an example). One can notice a slight difference between measured and modeled transmission, and this can slightly affect the value of the optical bandgap.

### 3.2. Optical Constants of Non-Stoichiometric IZTO in Wide Spectral Range

**Figure 2:**
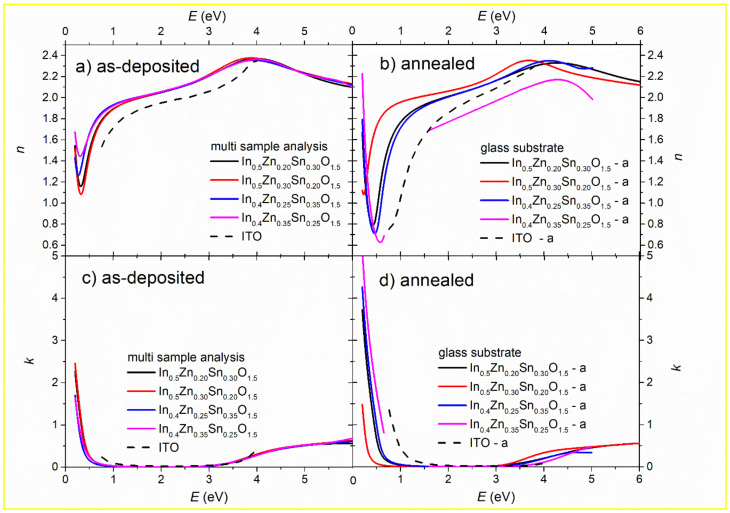
Spectral dependence of refractive index, *n*, and extinction coefficient, *k*, determined from spectroscopic ellipsometry for as-deposited (data from samples onto glass substrate and onto SiO_2_/Si substrate were fitted simultaneously) and annealed non-stoichiometric IZTO samples. Optical constants of indium tin oxide (ITO) [7] are added for comparison.

Figure 2 shows the determined spectral dependence of the refractive index, *n*, and extinction coefficient, *k*, for as-deposited and annealed non-stoichiometric IZTO samples. Data from multi-sample analysis (i.e., non-stoichiometric IZTO deposited onto the glass substrate and SiO_2_/Si substrate was fitted simultaneously) are displayed for as-deposited samples. Optical constants of ITO thin films deposited onto quartz substrates via RF sputtering with an ITO (99.99%) disc target of four inches in diameter from the literature [7] were added into Figure 2 for comparison. Sputtering of ITO was performed at an RF power of 150 W, for one hour, at substrate temperatures of room temperature and 350 °C. The distance between the target and the substrate was maintained at 10 cm, and Ar gas with a purity of 99.999%, at a flow rate of 30 sccm, was injected during sputtering. The base pressure of the chamber was less than 5.0 × 10^−6^ Torr, and the working pressure of the chamber during sputtering was about 1.5 × 10^−2^ Torr [7].

Only slight differences influenced by different substrates (especially in near–mid-infrared part spectra for Zn-rich samples) can be noticed (data not shown). Considering the similarity of obtained optical constants, annealed samples deposited onto the glass substrate were measured and analyzed only.

Refractive index, as well as extinction coefficient determined for as-deposited samples in the VIS–UV part of spectra, is nearly independent of composition (see Figure 2a,c) for photon energy *E* > 2 eV). Differences can be seen in near–mid infrared part spectra caused by different electrical resistivity of these samples and will be analyzed in detail later (see Figure 2a,c for *E* < 2 eV).

**Figure 3:**
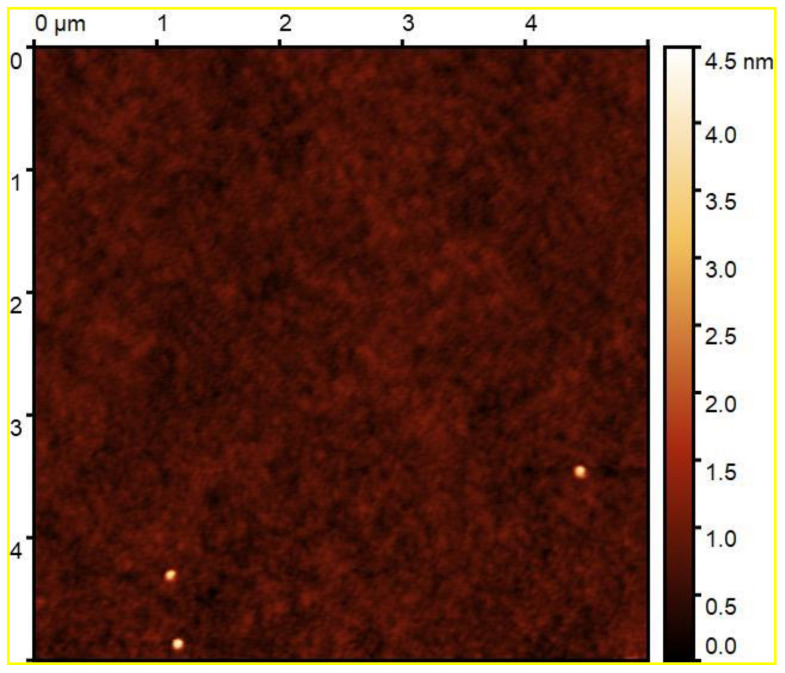
AFM scan of as-deposited In_0.5_Zn_0.30_Sn_0.20_O_1.5_ (IZTO25 Zn-rich) sample.

Noticeable differences in refractive index can be achieved by annealing with the conservation of relatively large transparent spectral window (compare Figure 2a,c with Figure 2b,d). Reported change of the refractive index has only a small effect on the transmittance of annealed samples of similar thickness with as-deposited ones (see Figure 3 in Reference [17]).

**Figure 4:**
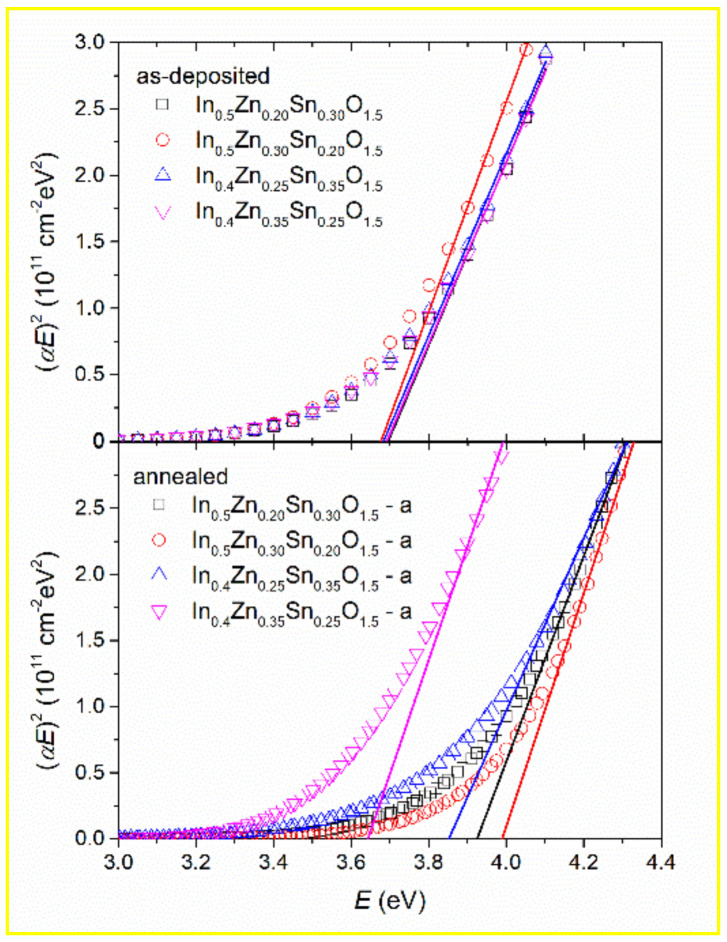
(*αE*)^2^ as a function of photon energy (symbols) for as-deposited and annealed non-stoichiometric IZTO samples obtained by using the parameterized semiconductor oscillator function (PSEMI) oscillator and its linear part described by Tauc model (solid lines).

It is worth mentioning that results from the PSEMI oscillator in the vicinity of short wavelength edge were compared with point by point fit (two unknown variables, *n* and *k*, are fitted for each wavelength, with fixed thickness). Good agreement between both results for all studied samples justifies the PSEMI oscillator as an appropriate candidate for the description of short wavelength edge of non-stoichiometric IZTO thin film samples. Moreover, correlations between parameters of the PSEMI oscillator were controlled and minimized by using a narrower spectral range for fit (vicinity of absorption edge). The similarity of the increase of the absorption coefficient, *α*, depicted in Figure 4 to that obtained from transmission measurement only (see, e.g., Figure 3 in Reference [16]) confirms the results obtained by ellipsometry.

Optical constants depicted in Figure 2 is used for the determination of different physical characteristics in the following sections.

### 3.3. Film Thickness and Surface Roughness

Measured thickness and surface roughness values of as-deposited and annealed films obtained using the aforementioned model by the best fit of ellipsometry data are summarized in Table 1.

The thickness of the as-deposited layers is about 80–90 nm. Notice that the substrate has no significant influence on the thickness of the as-deposited layer, thus indicating the good repeatability of the sputtering process. This fact is also supported by the similarity of optical constants of layers of the same composition deposited onto different substrates. Annealed layers of different thicknesses (of about 150 nm for IZTO30 films and of about 1 μm for IZTO25 films) were also analyzed.

Low surface roughness determined from spectroscopic ellipsometry and also from AFM (see Table 1) is a sign of the good optical quality of as-deposited layers. Considering results from AFM, even thicker annealed layers possess low surface roughness. In general, surface roughness of the as-deposited films onto the glass substrate is similar to that of the as-deposited ones onto the SiO_2_/Si substrate. A typical AFM scan of the as-deposited In_0.5_Zn_0.30_Sn_0.20_O_1.5_ (IZTO25 Zn-rich) sample can be seen in Figure 3.

### 3.4. Determination of Optical Bandgap

So-called “Tauc plot” [28], meaning dependence of (*αE*)^2^, where *α* = 4π*k*/*λ* and *λ* is wavelength on photon energy *E*, was constructed in order to determine the direct optical bandgap energy of non-stoichiometric IZTO samples and is depicted in Figure 4. The linear part of this dependence was used to estimate the optical bandgap (see lines in Figure 4). Determined optical bandgap values are summarized in Table 2.

As mentioned earlier, the optical constants of as-deposited samples in the vicinity of the absorption edge are very similar. Therefore, optical bandgaps of as-deposited samples are very similar even if the concentration of free carriers differs, as is seen later (see Figure 7). The bandgap energy values determined for annealed samples differ significantly from as-deposited samples, as well as among each other. The difference in optical bandgaps is very likely caused by different free carrier concentrations—see Figure 7 (Moss–Burstein effect [29,30]).

### 3.5. Free Electron Absorption in the NIR Part of Spectra

**Figure 5:**
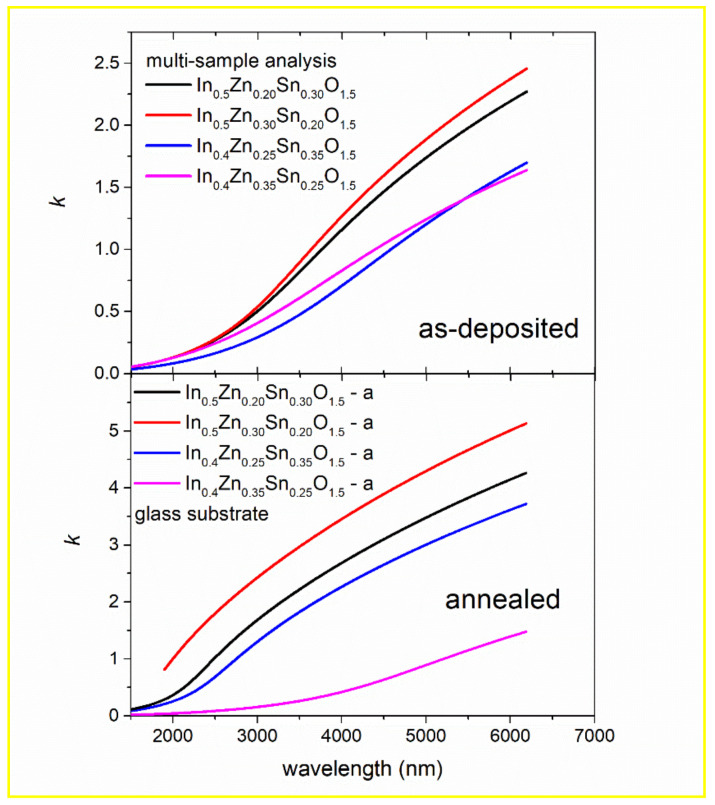
Extinction coefficient, *k*, as a function of wavelength in near–mid-infrared part of spectra for as-deposited and annealed non-stoichiometric IZTO samples obtained using Drude oscillator. Notice different scales for as-deposited and annealed samples.

The extinction coefficient of as-deposited and annealed non-stoichiometric IZTO samples as a function of wavelength is depicted in Figure 5. One can notice differences among the studied samples caused by the different specific electrical resistivity of these samples. A simple Drude oscillator used as a model dielectric function in the near–mid-infrared part of spectra (wavelength *λ* > 1500 nm) allows for the determination of specific electrical resistivity, *ρ*, and free carrier mean free time, *τ* (see Equation (3)):

*ε*_(DRUDE)_ = −*A*·*B*/(*E*^2^ + *B*·*E*) = −*ħ*^2^/(*ε*_0_·*ρ*·(*τ*·*E*^2^ + i·*ħ*·*E*)) (3)

where *E* is photon energy, *ε*_0_ permittivity of vacuum, *ħ* Planck constant, and parameters *A* and *B* can be obtained from the best fit of ellipsometry data.

The obtained results are summarized in Table 3 (with typical error ± 0.03 eV for parameter *A* and ± 0.002 eV for parameter *B*). Only slight differences for layers as-deposited onto different substrates can be noticed (more pronounced for Zn-rich samples).

**Figure 6:**
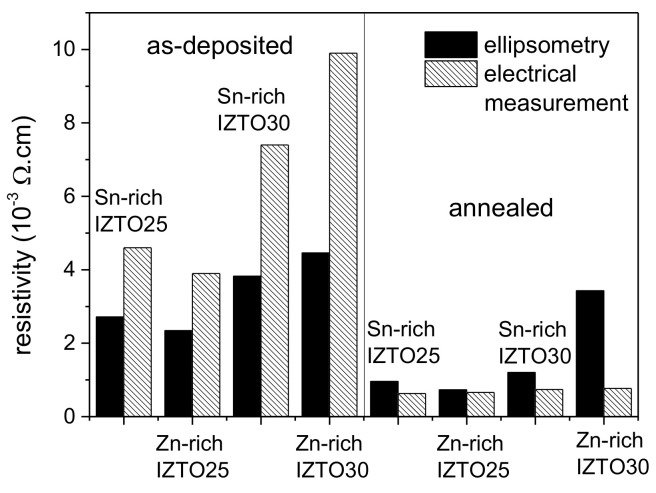
Comparison of specific electrical resistivity determined from near–mid-infrared part of spectroscopic ellipsometry spectra, using Drude oscillator and results from electrical measurements. Expected relative experimental error is about 1% in the case of electrical measurements and about 5% in the case of results obtained from ellipsometry measurements. (Sn-rich IZTO25 = In_0.5_Zn_0.20_Sn_0.30_O_1.5_, Zn-rich IZTO25 = In_0.5_Zn_0.30_Sn_0.20_O_1.5_, Sn-rich IZTO30 = In_0.4_Zn_0.25_Sn_0.35_O_1.5_ and Zn-rich IZTO30 = In_0.4_Zn_0.35_Sn_0.25_O_1.5_.)

Specific electrical resistivity obtained from the best fit of the ellipsometry data, using a simple Drude oscillator, can be compared with the results of the electrical measurements (see Figure 6). Although the agreement of absolute values is not perfect, the trends obtained from both methods correspond well to each other. One can notice the decrease of specific electrical resistivity, with the annealing confirmed by both methods. Specific electrical resistivity decreases by annealing for all samples consistently with the results of electrical measurements reported earlier [17]. Considering the simplicity of the used ellipsometry model (only a simple Drude oscillator was used in the near–mid-infrared wavelength range), the agreement is satisfactory.

**Figure 7:**
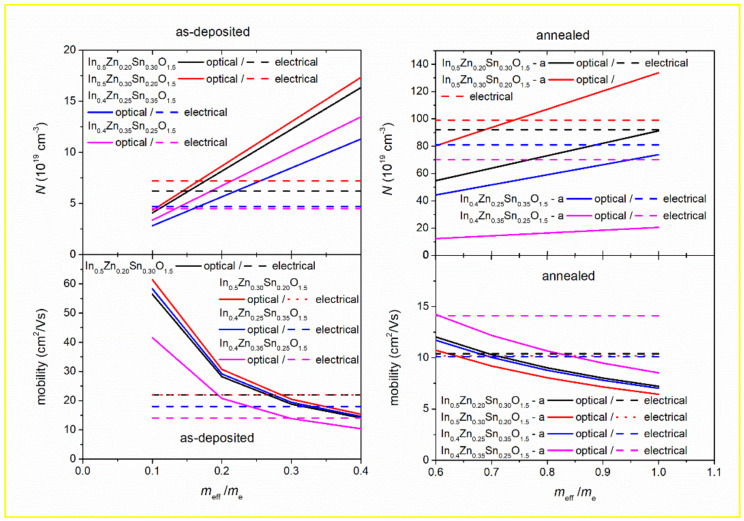
Dependence of free carrier concentration, *N*, and mobility, *μ*, calculated from simple Drude oscillator (solid lines) and results from electrical characterization (dashed and dotted lines) on effective mass (*m_e_* is mass of the free electron) for non-stoichiometric as-deposited and annealed IZTO films.

Moreover, specific electrical resistivity, *ρ*, can be calculated from the following relation:

*ρ* = 1/(*e*∙*N*∙*μ*) = m*/(*N*∙*e^2^*∙*τ*) (4)

and therefore, free carrier concentration, *N*, and mobility, *μ*, can be determined from a simple Drude oscillator, using effective electron mass, m*, and charge, *e*. Dependence of free carrier concentration, *N*, and mobility, *μ*, on effective mass (for m* from 0.1·*m*_e_ to *m*_e_, where *m*_e_ is the mass of a free electron) is depicted in Figure 7 and compared with results of electrical measurement (Hall effect and four-point sheet resistance measurements) for all samples.

Considering the experimental error of both methods (1% for electrical measurement and 5% for results obtained from ellipsometry), the agreement between free carrier concentration and mobility obtained from the best fit of ellipsometry data using simple Drude oscillator and determined from electrical measurement can be achieved if 0.1·*m*_e_ < m* < 0.3·*m*_e_ for as-deposited samples (see Figure 7 (left column)). This interval is close to the effective mass of IZTO reported in the literature (~0.2·*m*_e_) [31].

For annealed samples, the agreement between the results obtained from ellipsometry and from electrical measurements is achieved if m* > 0.6·*m*_e_ (see Figure 7, right column), suggesting a possible increase of the effective mass, which is very likely connected with the change in the electronic structure and/or different degree of amorphousness induced by annealing.

From an electrical point of view, the idea of stoichiometric IZTO is based on co-substitution of In^3+^ in In_2_O_3_ by Sn^4+^ and Zn^2+^ from SnO_2_ and ZnO described by the following equation:

SnO_2_ + ZnO → (In_2_O_3_) → Sn_In_^+^ + Zn_In_^−^ + 3O_O_. (5)

In spite of Equation (5), the excess of Sn^4+^ and Zn^2+^ should produce donors and acceptors by substitution of In^3+^ in In_2_O_3_. In oxide semiconductors, free carrier generation is connected to oxygen vacancies (see Equations (6) and (7)):

2SnO_2_ → (In_2_O_3_) → 2Sn_In_^+^ + 3O_O_ + O_2_(g)/2 + 2e^−^. (6)

2ZnO → (In_2_O_3_) → 2Zn_In_^−^ + 2O_O_ + V_O_^2+^. (7)

It is known that the incorporation of oxygen leads to the decrease in oxygen vacancies in the films, and hence to the drop in the carrier concentration [32], as follows:

O_2_(g)/2 + V_O_^2+^ + 2e^−^ → O_O_. (8)

Differences in free carrier concentration caused by different compositions can, therefore, explain the change of free carrier concentration (see Figure 7) and subsequently different specific electrical resistivity (see Table 3 and Figure 6). It is noted, however, that several defect reactions, including the ones shown above, are competing among them during the sample processing step, and thereby the resulting electron carrier concentration and resistivity values are usually not predictable and strongly influenced by the changes in sample preparation condition. Nonetheless, since the condition is fixed in this study, it is expected that electrical transport parameters such as free carrier concentration, mobility, and specific electrical resistivity determined from near–mid-infrared absorption spectra would be closely related to the ones obtained from electrical characterization, such as the Hall effect and four-point sheet resistance measurements.

It is worth mentioning that results obtained by using Drude and Lorentz oscillators in near–mid-infrared wavelength range were compared with point-by-point fit (two unknown variables, *n* and *k*, are fitted for each wavelength with fixed thicknesses)—data not shown. One can notice that results obtained by Drude and Lorentz oscillators are practically indistinguishable. Although the agreement between the results from point-by-point fit and Drude oscillator is satisfactory, still one can notice disagreement especially for higher wavelengths (MIR), which can cause differences between specific electrical resistivity obtained from electrical and optical measurements presented in Figure 6.

### 3.6. Results from SEM

**Figure 8:**
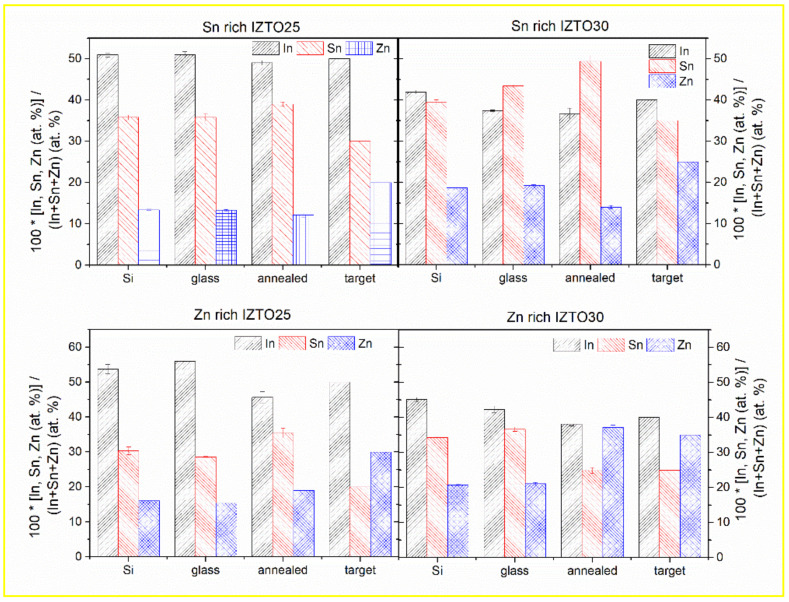
Actual composition of In, Sn, and Zn for non-stoichiometric IZTO films deposited onto the glass and SiO_2_/Si substrates and annealed non-stoichiometric IZTO films onto the glass substrates obtained by EDS, together with expected (target) composition. Sum of at.% of In, Sn, and Zn is taken as 100.

**Figure 9:**
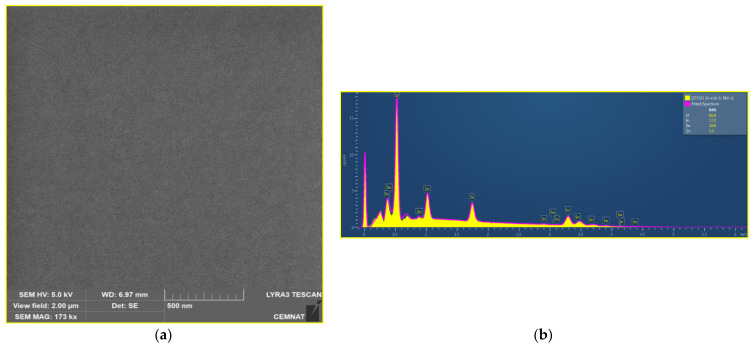
(**a**) SEM micrograph (left part) together with (**b**) example of EDS spectra (right part) of In_0.5_Zn_0.30_Sn_0.20_O_1.5_ (Zn-rich IZTO25) as-deposited onto SiO_2_/Si substrate.

The actual composition of the layers was measured by EDS, at three different places, and the results are summarized in Figure 8, together with the composition of the ceramic sputtering target (where sum of at.% of In, Sn, and Zn is taken as 100). An example of EDS spectra can be found in Figure 9, where the Si peak from substrate and O peak from layer and substrate are also visible). For Si/glass substrates, more pronounced differences in the composition can be seen for Zn-rich as-deposited layers. These differences in composition might be a reason for differences in optical constants in the near–mid-infrared part of the spectra, as well as in the free carrier concentration and mobility (see Figure 7) and subsequently also in specific electric resistivity (see Table 3). One can also notice a difference in composition between annealed and as-deposited layers. Considering that the penetration depth of EDS measurement is about 200 nm and the thicknesses of the majority of the layers are lower than 150 nm, the experimental error cannot be neglected.

For the majority of the samples, the SEM images look very similar (see Figure 9 as an example), suggesting no significant change of the surface structure induced, neither by the difference in the substrate nor by annealing.

## 4. Conclusions

Ellipsometry parameters of four non-stoichiometric as-deposited and annealed IZTO thin films with various In:Zn:Sn composition were studied. A multi-sample analysis including samples prepared onto the SiO_2_/Si and the glass substrate, together with the analysis of the layer deposited onto each substrate separately, was conducted. The following parameters/trends were determined:

1. The thickness of the studied films, as well as surface roughness, was determined.
2. The specific electrical resistivity calculated by using a simple Drude oscillator in the near–mid-infrared part of spectra agreed well with the results obtained from electrical measurements.
3. A decrease of electrical resistivity induced by annealing reported earlier was confirmed by electrical, as well as optical, measurements.
4. The comparison of free carrier concentration and free carrier mobility obtained using a simple Drude oscillator and the results from electrical measurement suggests an increase of effective mass induced by annealing.
5. The shift of direct energy bandgap estimated from the Tauc plot due to the change of free carrier concentration was observed, i.e., the Moss–Burstein effect, which was even more pronounced for annealed samples.
6. The negligible influence of a different substrate on thickness, surface roughness, refractive index, and extinction coefficient for as-deposited non-stoichiometric IZTO films was found, suggesting very good repeatability of the sputtering process.
7. The negligible extinction coefficient and the similarity of the refractive index for the visible part of the spectrum allow the utilization of as-deposited non-stoichiometric IZTO films and hence the decrease of In content without the substantial change of the optical properties.
8. The change of the refractive index of non-stoichiometric IZTO layers after annealing does not significantly influence their high transparency, and a wide transparent window of non-stoichiometric IZTO layers is preserved after annealing.

In this article, the capabilities of spectroscopic ellipsometry were examined as an alternative tool to characterize the electrical properties of the as-deposited and annealed non-stoichiometric IZTO films deposited by RF magnetron sputtering. To our knowledge, this is the first time when spectroscopic ellipsometry was applied to these samples. The good agreement of the results obtained by spectroscopic ellipsometry with other methods (transmission measurements, electrical measurements, and AFM and SEM measurements) allows us to conclude that the spectroscopic ellipsometry can serve as a valuable tool for further research on transparent conductive oxide thin films.

## Figures and Tables

**Table 1:** Thickness, surface roughness, and mean square error (MSE) of non-stoichiometric IZTO films deposited onto glass and SiO_2_/Si substrates and annealed non-stoichiometric IZTO films onto glass substrates obtained by the best fit of ellipsometry data together with surface roughness obtained from atomic force microscopy (AFM).

Sample	Thickness of Samples (on Glass) (nm)	Thickness of Samples (on Si) (nm)	Surface Roughness (on Glass) (nm)	Surface Roughness (on Si) (nm)	MSE Glass/Si Substrate	AFM Surface Roughness Glass/Si Substrate (nm)
In_0.5_Zn_0.20_Sn_0.30_O_1.5_ (Sn-rich IZTO25)	(88.3 ± 0.3) ^1^ (1224 ± 2) ^2^	(88.5 ± 0.2) ^1^	(4.9 ± 0.3) ^1^ (9.9 ± 0.3) ^2^	(2.6 ± 0.3) ^1^	(11.2/4.1) ^1^ (9.9) ^2^	(0.10/0.18) ^1^ (0.38) ^2^
In_0.5_Zn_0.30_Sn_0.20_O_1.5_ (Zn-rich IZTO25)	(82.6 ± 0.3) ^1^ (1074 ± 6) ^2^	(83.3 ± 0.3) ^1^	(2.3 ± 0.3) ^1^ (6.6 ± 0.9) ^2^	(2.8 ± 0.3) ^1^	(10.9/4.3) ^1^ (35.0) ^2^	(0.14/0.13) ^1^ (1.41) ^2^
In_0.4_Zn_0.25_Sn_0.35_O_1.5_ (Sn-rich IZTO30)	(91.2 ± 0.3) ^1^ (121.1 ± 0.3) ^2^	(90.4 ± 0.3) ^1^	(1.8 ± 0.3) ^1^ (3.8 ± 0.3) ^2^	(2.1 ± 0.3) ^1^	(11.4/3.7) ^1^ (3.2) ^2^	(0.17/0.14) ^1^ (0.07) ^2^
In_0.4_Zn_0.35_Sn_0.25_O_1.5_ (Zn-rich IZTO30)	(79.3 ± 0.3) ^1^ (149.0 ± 0.3) ^2^	(79.9 ± 0.3) ^1^	(2.9 ± 0.3) ^1^ (4.6 ± 0.3) ^2^	(2.6 ± 0.3) ^1^	(11.0/3.7) ^1^ (1.6) ^2^	(0.25/0.14) ^1^ (0.11) ^2^

^1^ As-deposited; ^2^ annealed.

**Table 2:** Optical bandgap of as-deposited and annealed non-stoichiometric IZTO layers determined from Tauc plot.

Sample/*E*_g_^opt^ (eV)	As-Deposited/Multi-Sample Analysis	Annealed/Glass Substrate
In_0.5_Zn_0.20_Sn_0.30_O_1.5_ (Sn-rich IZTO25)	3.70	3.92
In_0.5_Zn_0.30_Sn_0.20_O_1.5_ (Zn-rich IZTO25)	3.68	3.95
In_0.4_Zn_0.25_Sn_0.35_O_1.5_ (Sn-rich IZTO30)	3.68	3.85
In_0.4_Zn_0.35_Sn_0.25_O_1.5_ (Zn-rich IZTO30)	3.69	3.64

**Table 3:** Parameters *A* (typical error ± 0.03 eV) and *B* (typical error ± 0.002 eV) for simple Drude oscillator (see Equation (3)) obtained from the best fit of ellipsometry data and calculated specific electrical resistivity, *ρ*, and free carrier mean free time, *τ* (see Equation (3)), for non-stoichiometric IZTO films deposited onto glass and SiO_2_/Si substrates and annealed non-stoichiometric IZTO films onto glass substrates.

Sample	*A*_Drude_ (eV)	*B*_Drude_ (eV)	*ρ* (mΩ⋅cm)	*τ* (fs)
In_0.5_Zn_0.20_Sn_0.30_O_1.5_ (Sn-rich IZTO25)	(2.75/2.74) ^1^ (7.84) ^2^	(0.205/0.199) ^1^ (0.160) ^2^	(2.9/2.7) ^1^ (1.0) ^2^	(2.9/3.3) ^1^ (4.0) ^2^
In_0.5_Zn_0.30_Sn_0.20_O_1.5_ (Zn-rich IZTO25)	(3.17/3.25) ^1^ (10.26) ^2^	(0.188/0.186) ^1^ (0.180) ^2^	(2.7/2.3) ^1^ (0.7) ^2^	(3.1/3.5) ^1^ (3.6) ^2^
In_0.4_Zn_0.25_Sn_0.35_O_1.5_ (Sn-rich IZTO30)	(1.96/1.97) ^1^ (6.18) ^2^	(0.199/0.199) ^1^ (0.165) ^2^	(3.8/3.8) ^1^ (1.2) ^2^	(3.3/3.3) ^1^ (4.0) ^2^
In_0.4_Zn_0.35_Sn_0.25_O_1.5_ (Zn-rich IZTO30)	(1.67/1.69) ^1^ (2.09) ^2^	(0.278/0.280) ^1^ (0.136) ^2^	(5.1/4.4) ^1^ (3.4) ^2^	(2.4/2.4) ^1^ (4.9) ^2^

^1^ As-deposited multi-sample analysis/glass substrate; ^2^ annealed.

## Data Availability

The data presented in this study are openly available in The Digital Library of the University of Pardubice.

## References

[1] Lee J.-A., Lee J.-H., Heo Y.-W., Kim J.-J., Lee H.Y. (2012). Characteristics of Sn and Zn co-substituted In_2_O_3_ thin films prepared by RF magnetron sputtering. Curr. Appl. Phys..

[2] Damisih I., Ma H.C., Finanda F., Kim J.-J., Lee H.Y. (2012). Effect of Composition on Transparent Conducting Indium Zinc Tin Oxide Thin Films Deposited by RF Magnetron Sputtering. J. Nanoelectron. Optoelectron..

[3] Ma H.C., Damisih I., Putri M., Cheon J.H., Kim J.H., Lee H.Y. (2012). The Effect of Post-annealing Treatment on the Characteristics of a Dye-sensitized Solar Cell with an Indium Zinc Tin Oxide Electrode. J. Korean Phys. Soc..

[4] Minami T. (2008). Present status of transparent conducting oxide thin-film development for Indium-Tin-Oxide (ITO) substitutes. Thin Solid Film..

[5] Alam M.J., Cameron D.C. (2000). Optical and electrical properties of transparent conductive ITO thin films deposited by sol-gel process. Thin Solid Film..

[6] Badawy W.A., Afify H.H., Elgiar E.M. (1990). Optical and Photovoltaic Characteristics of In-Modified SnO_2_ Thin Films. J. Electrochem. Soc..

[7] Kang M., Kim I., Chu M., Kim S.W., Ryu J.-W. (2011). Optical Properties of Sputtered Indium-tin-oxide Thin Films. J. Korean Phys. Soc..

[8] Boussoum O., Belkaid M.S., Renard C., Halais G., Farhati F. (2019). Effect of the annealing gas and RF power sputtering in the electrical, structural and optical properties of ITO thin films. J. Nano Electron. Phys..

[9] Rasheed M., Barillé R. (2017). Optical constants of DC sputtering derived ITO, TiO_2_ and TiO_2_:Nb thin films characterized by spectrophotometry and spectroscopic ellipsometry for optoelectronic devices. J. Non-Cryst. Solids.

[10] Tamanai A., Dao T.D., Sendner M., Nagao T., Pucci A. (2017). Mid-infrared optical and electrical properties of indium tin oxide films. Phys. Status Solidi A.

[11] Damisih I., Ma H.C., Yoon D.J., Kim J.-J., Lee H.Y. (2011). Transparent Conductive Indium Zinc Tin Oxide Thin Films for Solar Cell Applications. J. Nanoelectron. Optoelectron..

[12] Carreras P., Antony A., Roldán R., Nos O., Frigeri P.A., Asensi J.M., Bertomeu J. (2010). Transparent conducting thin films by co-sputtering of ZnO-ITO targets. Phys. Status Solidi C.

[13] Ko Y.-D., Kim J.-Y., Joung H.-C., Ahn S.-H., Jang K.-S., Lee Y.-J., Yi J. (2013). Low temperature deposited transparent conductive ITO and IZTO films for flat panel display applications. J. Ceram. Process. Res..

[14] Putri M., Koo C.Y., Lee J.-A., Kim J.-J., Lee H.Y. (2014). Transparent conducting indium zinc tin oxide thin films with low indium content deposited by radio frequency magnetron sputtering. Thin Solid Film..

[15] Noviyana I., Lestari A.D., Putri M., Won M.-S., Bae J.-S., Heo Y.-W., Lee H.Y. (2017). High Mobility Thin Film Transistors Based on Amorphous Indium Zinc Tin Oxide. Materials.

[16] Kim K.H., Putri M., Lee H.J., Koo C.Y., Lee J.-A., Kim J.-J., Lee H.Y. (2015). Non-Stoichiometric Indium Zinc Tin Oxide Thin Films Prepared by RF Magnetron Sputtering. J. Nanoelectron. Optoelectron..

[17] Putri M., Kim K.H., Koo C.Y., Lee J.-A., Kim J.-J., Baikie I.D., Grain A.C., Lee H.Y. (2017). Effect of Annealing Treatment on the Properties of Stoichiometric Indium Zinc Tin Oxide (IZTO) Thin Films. J. Nanoelectron. Optoelectron..

[18] Lee H.J., Noviyana I., Koo C.Y., Lee J.-A., Kim J.-J., Lestari A.D., Jeong Y., Lee Y., Lee H.Y. (2017). Organic Photovoltaics with Non-Stoichiometric InZnSnO Thin Film Cathodes. J. Nanosci. Nanotechnol..

[19] Herzinger C.M., Johs B., McGahan W.A., Woollam J.A., Paulson W. (1998). Ellipsometric determination of optical constants for silicon and thermally grown silicon dioxide via a multi-sample, multi-wavelength, multi-angle investigation. J. Appl. Phys..

[20] Pujar P., Gandla S., Singh M., Gupta B., Tarafder K., Gupta D., Nohd Y.-Y., Mandal S. (2017). Development of low temperature stoichiometric solution combustion derived transparent conductive ternary zinc tin co-doped indium oxide electrodes. RSC Adv..

[21] J.A. Woollam Co. Inc (2000). Guide to Using WVASE32 Software for Spectroscopic Ellipsometry Data Acquisition and Analysis.

[22] Ihn Y.S., Kim T.J., Ghong T.H., Kim Y.D., Aspnes D.E., Kossut J. (2004). Parametric modeling of the dielectric functions of Cd_1-x_Mg_x_Te alloy films. Thin Solid Film..

[23] Johs B., Herzinger C.M., Dinan J.H., Cornfeld A., Benson J.D. (1998). Development of a parametric optical constant model for Hg_1-x_Cd_x_Te for control of composition by spectroscopic ellipsometry during MBE growth. Thin Solid Film..

[24] Tiwald T.E., Thompson D.W., Woollam J.A., Paulson W., Hance R. (1998). Application of IR variable angle spectroscopic ellipsometry to the determination of free carrier concentration depth profiles. Thin Solid Film..

[25] Bruggeman D.A.G. (1935). Berechnung verschiedener physikalischer Konstanten von heterogenen Substanzen. I. Dielektrizitätskonstanten und Leitfähigkeiten der Mischkörper aus isotropen Substanzen. Ann. Phys..

[26] Janicek P., Niang K.M., Mistrik J., Palka K., Flewitt A.J. (2017). Spectroscopic ellipsometry characterization of ZnO:Sn thin films with various Sn composition deposited by remote-plasma reactive sputtering. Appl. Surf. Sci..

[27] Janicek P., Slang S., Palka K., Vlcek M. (2017). Spectroscopic ellipsometry characterization of spin-coated Ge_25_S_75_ chalcogenide thin films. Pure Appl. Chem..

[28] Tauc J. (1974). Amorphous and Liquid Semiconductors.

[29] Burstein E. (1954). Anomalous Optical Absorption Limit in InSb. Phys. Rev..

[30] Moss T.S. (1954). The Interpretation of the Properties of Indium Antimonide. Proc. Phys. Soc..

[31] Lu Y.-B., Li Y.H., Ling Z.C., Cong W.-Y., Zhang P., Xin Y.Q., Yang T.L. (2016). Geometric, electronic and optical properties of zinc/tin codoped In_2_O_3_ modulated by the bixbyite/corundum phase transition. J. Phys. D Appl. Phys..

[32] Han Y., Kim D., Cho J.-S., Koh S.-K., Song Y.S. (2001). Tin-doped indium oxide (ITO) film deposition by ion beam sputtering. Sol. Energy Mater. Sol. Cells.

